# Diagnosis of early mild cognitive impairment using a multiobjective optimization algorithm based on T1-MRI data

**DOI:** 10.1038/s41598-022-04943-3

**Published:** 2022-01-19

**Authors:** Jafar Zamani, Ali Sadr, Amir-Homayoun Javadi

**Affiliations:** 1 School of Electrical Engineering, Iran University of Science and Technology, Narmak, Tehran, Iran; 2 School of Psychology, Keynes College, University of Kent, Canterbury, UK; 3 School of Rehabilitation, Tehran University of Medical Sciences, Tehran, Iran

**Keywords:** Diagnostic markers, Image processing, Alzheimer's disease, Alzheimer's disease

## Abstract

Alzheimer’s disease (AD) is the most prevalent form of dementia. The accurate diagnosis of AD, especially in the early phases is very important for timely intervention. It has been suggested that brain atrophy, as measured with structural magnetic resonance imaging (sMRI), can be an efficacy marker of neurodegeneration. While classification methods have been successful in diagnosis of AD, the performance of such methods have been very poor in diagnosis of those in early stages of mild cognitive impairment (EMCI). Therefore, in this study we investigated whether optimisation based on evolutionary algorithms (EA) can be an effective tool in diagnosis of EMCI as compared to cognitively normal participants (CNs). Structural MRI data for patients with EMCI (n = 54) and CN participants (n = 56) was extracted from Alzheimer’s disease Neuroimaging Initiative (ADNI). Using three automatic brain segmentation methods, we extracted volumetric parameters as input to the optimisation algorithms. Our method achieved classification accuracy of greater than 93%. This accuracy level is higher than the previously suggested methods of classification of CN and EMCI using a single- or multiple modalities of imaging data. Our results show that with an effective optimisation method, a single modality of biomarkers can be enough to achieve a high classification accuracy.

## Introduction

Dementia is the greatest healthcare issue in the twenty-first century causing cognitive decline, disabilities and finally death to an aging population^1^. AD is the most frequent neurodegenerative disease and has received much public attention due to putting excessive costs on society and a significant burden on family members^2^. Despite vast investigations, there is still no reliable cure for AD^3^, mainly because the physiology and etiopathology of AD still remains unclear due to its multifactorial nature^4^. Identifying AD at an early phase is essential to ensuring proper care of patients and also to developing and testing new treatment approaches. Mild cognitive impairment (MCI) usually represents a transitional phase between normal aging and clinically probable AD^5,6^. Structural changes in the brain have been shown to be one of the earliest biomarkers that can be used in the diagnosis of AD^7,8^, such as atrophy. Neuroimaging tools, in particular structural magnetic resonance imaging (sMRI), are used for measures of atrophy, especially because the atrophic process occurs earlier than the appearance of behavioural amnestic symptoms^9,10^.

The pattern of structural changes, however, is complicated; atrophy does not occur uniformly across all the brain. Therefore, many researchers are investigating to identify which brain areas are more reliable in diagnosis of MCI and AD^9^. Hippocampal volume loss, in particular, has been shown to be an indication of AD^11,12^, however, even the subfields of the hippocampus do not shrink uniformly, perhaps due to their specialisation^13,14^. Identification of the brain areas that are most affected by MCI is even harder due to the changes being smaller^10,15,16^. Therefore, it is important to find methods that can successfully classify those with MCI.

To identify profound brain changes induced by the atrophic process in MRI data, various computer aided diagnosis (CAD) approaches are used for the early diagnosis of AD, as well as MCI^5^. CAD methods in MRI usually contain three fundamental components: (1) segmentation, (2) extraction of the features (e.g., volume and percentage), and (3) classification^17–19^. For segmentation, with the development of semi- and fully-automated segmentation methods, it has now become easier and faster to segment the cortex as well as the hippocampus^20,21^. Hippocampus subfield Segmentation (HIPS)^22^, volBrain^23^, and Computational Anatomy Toolbox (CAT)^24,25^ are some of the commonly used fully-automated methods. The segmented brain areas are used as features in the classification methods^26,27^.

The number of extracted parameters, however, are quite large which leads to complication of the classification methods. CAD methods typically suffer from the challenge of overfitting, due to the very high dimensionality of extracted features compared to the number of data points for model training. To overcome this challenge, dimension reduction of the features is an essential step for selecting the optimal subset of features. The essential problem of the feature selection algorithms is finding the relevant subset of features that yields high performance^28^. Evolutionary algorithms (EAs) offer an effective optimisation method, especially in large search spaces. For example, methods such as nondominated sorting genetic algorithm II (NSGA-II) have the computational complexity required to select features in multidimensional classification applications^29^. The aim of these methods is finding the optimum number of features with minimum classification error^30^.

This paper investigates the application of EAs using nondominated sorting NSGA-II, which is a very novel method of EA, as well as more established methods of genetic algorithm (GA), ant colony optimisation (ACO), simulated annealing (SA) and particle swarm optimisation (PSO) in classification of early stages of AD and cognitively normal (CN) subjects using brain areas volumetric information of the T1-MRI data. We compared the results of EAs with the results based on statistical feature selection methods. We used three automated segmentation methods, volBrain and CAT for segmentation of the whole brain, and HIPS for segmentation of subfields of the hippocampus. For the classification, we used multi-layer perceptron artificial neural network (ANN).

## Results

**Figure 1:**
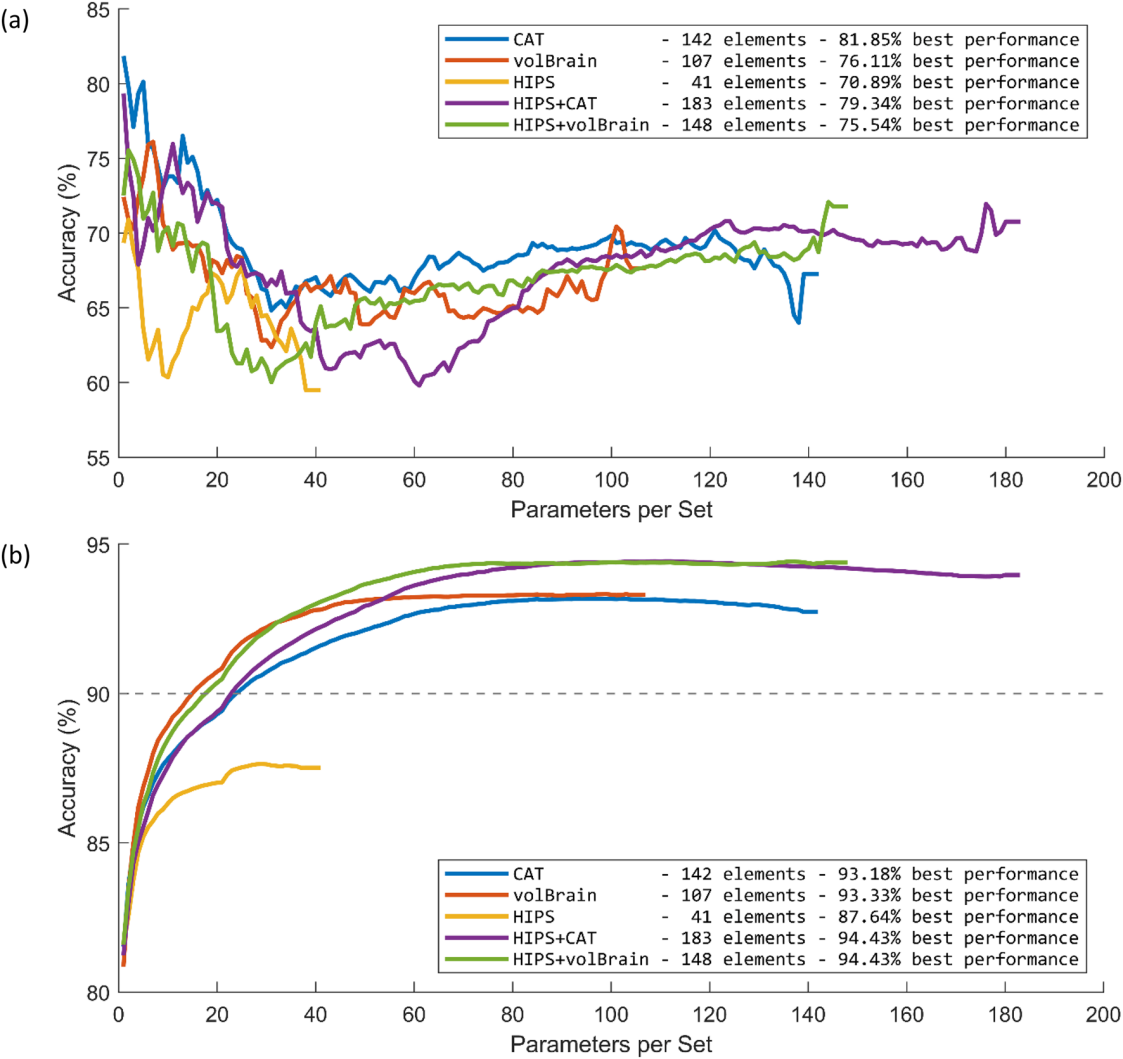
Performance of the proposed algorithms for the five datasets. (**a**) Performance of the optimisation based on the statistical comparison of the two groups of EMCI and CN. (**b**) Average performance of the five evolutionary algorithms (EA).

Using CAT and volBrain we extracted 142 and 107 parameters indicating the volume, percentage and asymmetry between the two hemispheres across the whole brain. We also used HIPS to segment the hippocampus and extracted 41 parameters indicating the volume, percentage and asymmetry between the two hemispheres. Combining CAT and HIPS, as well as, volBrain and HIPS, we generated two additional sets of parameters. Due to non-normal distribution of the parameters, we used non-parametric two independent-sample t-tests to compare the parameters from the EMCI and CN groups. Details of these comparisons are listed in Supplementary Tables 1–3. Twenty-eight out of 142 parameters (19.71%) in CAT showed significant differences between the two groups. Some of these brain areas included bilateral pre- and post-central gyri, and the right inferior frontal gyrus that showed significantly smaller size in the EMCI group. Interestingly, some brain areas such as hippocampus and amygdala were not significantly different. No volBrain and HIPS parameters were significantly different between the two groups after false discovery rate (FDR) correction for multiple comparison^31,32^. Only a few parameters showed a trend towards significance, such as asymmetry between left and right hemispheres of cerebrum and CA1 in volBrain and HIPS, respectively. The parameters were sorted based on the p-values to investigate whether the multi-layer perceptron ANN can classify the two groups. Figure 1a shows the performance of this method for different numbers of included parameters for the five datasets. The performance of this method was quite poor with the best accuracy of 81.85%.

We used five EA methods to investigate whether optimisation algorithms that were specifically designed for searches in large search-spaces are effective in the classification of the two groups. Figure 1b shows the average performance of the five EA methods. This plot shows that the EA methods achieved high classification accuracy of almost 93% for the volBrain and CAT, and almost 94% for the combination of volBrain and CAT with HIPS. Interestingly, the optimisation based on HIPS parameters alone achieved a lower accuracy level of almost 87%.

**Figure 2:**
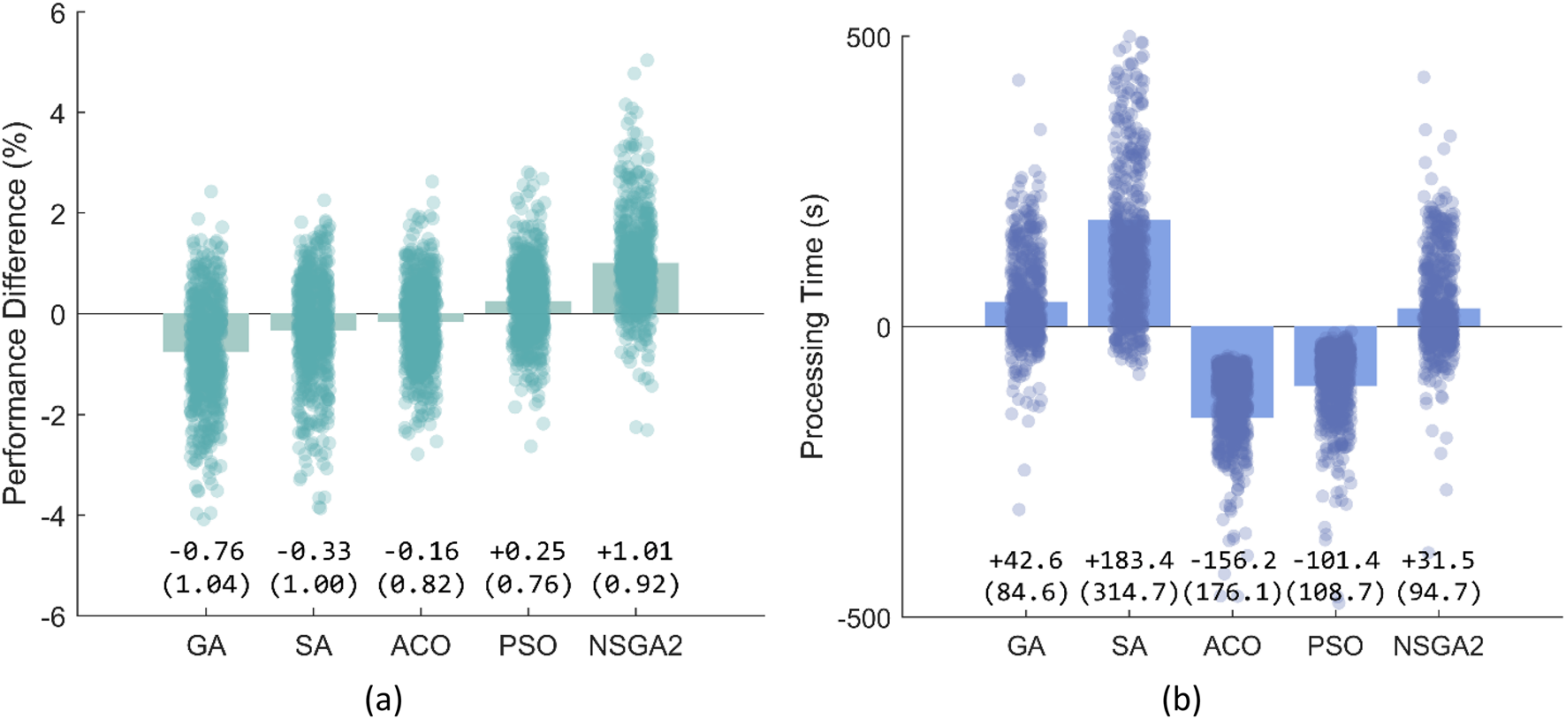
Comparison of the five evolutionary algorithms (EA) in terms of accuracy (**a**) and processing time (**b**). Values reported are mean (SD) difference compared to the average of the five EAs. *GA* Genetic algorithm, *NSGA2* nondominated sorting genetic algorithm II, *ACO* ant colony optimisation, *SA* simulated annealing, *PSO* particle swarm optimisation.

Comparison between the EA methods showed quite similar performance across the method. NSGA-II, however, achieved the best accuracy, 1.01% more accurate than the average. ACO was the fastest algorithm outperforming other algorithms for about 156 s per optimisation cycle. See Fig. 2 for details.

To investigate the brain areas that are most involved in the recognition of CN and EMCI, we extracted the list of the five most indicative brain areas based on the number of times that they appeared in the 100 simulations for the five EA’s. For the complete list of the brain areas, please refer to Table 1.

**Table 1:** Summary of the five most indicative brain areas in the classification of CN and EMCI.

Segmentation	Brain areas	Segmentation	Brain areas
CAT	Occipital fusiform gyrus	CAT + HIPS	4th ventricle
	Frontal pole		Occipital fusiform gyrus
	4th ventricle		Frontal pole
	Lateral ventricle		Subiculum
	CLCVL		SR–SL–SM
volBrain	Caudate	volBrain + HIPS	Caudate
	Putamen		Putamen
	Cerebrum		Lateral ventricles
	Accumbens		Hippocampus
	Lateral ventricles		Accumbens
HIPS	SR–SL–SM		
	Hippocampus		
	CA2-CA3		
	CA1		
	CA4-DG		

*CLCVL* Cerebellar lobule cerebellar vermal lobules, *SR–SL–SM* strata radiatum/lacunosum/moleculare.

To investigate whether our method is successful in classification of CN participants from those with AD, we extracted data for a set of 54 participants with AD and applied our algorithm to this data. Our analysis showed a very high average classification accuracy of 95%. See Supplementary Table 5 for details of the demographics of the participants and Supplementary Table 6 for the breakdown of the classification accuracy for different segmentation and classification methods.

## Discussion

Using three automated methods, we segmented the whole brain (volBrain and CAT) and the subfields of the hippocampus (HIPS) using T1-weighted MRI data of EMCI (n = 54) and CN (n = 56) individuals. Our proposed optimisation method based on evolutionary algorithms (EA) achieved a higher classification accuracy than the conventional statistical methods. There was no significant difference between the EA algorithms in terms of performance. Classification based on hippocampus subfields was poorer than whole brain subfields. Combination of the whole brain and hippocampus subfields, however, improved the classification accuracy.

While classification methods have been effective in diagnosing AD, their success the in diagnosis of MCI has been very limited. This is due to smaller and more minute changes in MCI as compared to AD. Therefore, recognition of changes from healthy to mildly cognitively impaired is quite difficult. Table 2 compares the results of our study with the previous studies using data from ADNI on classification of EMCI and CN. It shows that our approach achieved the best performance accuracy with 94.4% classification accuracy. In addition to superior performance, our approach has multiple advantages over other studies: it is based on only one biomarker, (2) it is highly interpretable, (3) high accuracy levels base on relatively low number of participants, and (4) the preprocessing was done using fully automated pipelines.

**Table 2:** Summary of the studies using ADNI dataset for classification of patients with early mild cognitive impairment (EMCI) and cognitively normal (CN) participants.

Reference	Data	Subjects (N)	EMCI vs CN (%)
Guerrero et al.^33^	MRI	363	65.0
Prasad et al.^34^	DWI	124	59.2
Jie et al.^35^	MRI + fMRI	106	78.2
Wee et al.^36^	MRI	614	51.8
Lee et al.^37^	MRI + PET + DTI	128	70.5
Fang et al.^38^	MRI + PET	548	79.2
Kang et al.^39^	MRI + DTI	120	94.2
Kam et al.^40^	MRI + fMRI	97	76.0
Yang et al.^41^	MRI + fMRI	58	82.7
Proposed method	MRI	110	94.4

*DTI* diffusion tensor imaging, *DWI* diffusion-weighted imaging, *PET* positron emission therapy.

In this study we used only structural MRI (T1-weighted MRI) images which is one of the most commonly used neuroimaging methods in clinical settings. One important aspect of structural MRI is that is intendent of participant’s behaviour, which might rely heavily on general cognitive ability and mood of the participant at the time of measurement. Reliance on a single neuroimaging modality that is independent of the patient’s behaviour is an advantage over other classification methods that use two or more neuroimaging methods (e.g., DTI and PET, see for example^37–39^) that occasionally rely on participants’ response to different stimuli (e.g., fMRI, see for example^35,40,41^): it reduces the burden on the patient and reduces the costs.

Our method was based on volumetric data: brain images were segmented into smaller brain areas over the cortex (volBrain and CAT) as well as hippocampus (HIPS) using standardised brain atlases. Therefore, the individual components involved in the optimisation algorithms reflect the size of each brain area, which is extremely interpretable^26^. This is in contrast to optimisation methods based on deep neural networks and support vector machines that are mostly considered as black boxes^42^. Interpretability enables us to have a better understanding of the mechanisms underlying diseases^43,44^. For example, a closer look at the data showed that atrophy in the amygdala and caudate are better indicators of EMCI as compared to atrophy in the precuneus, and pre- and post-central gyri. In addition, while overall hippocampus volume was significantly different between the two groups, none of its subfields showed significant difference between CN and EMCI^13,14,45,46^. This is of great interest as although hippocampus atrophy is typically considered as the hallmark of AD^12,47^, brain areas in the rest of the cortex were better indicators of EMCI. This is an important finding as it highlights that changes in cognitive domains that are less reliant on the hippocampus, such as executive functions, are better indicators of behavioural outcomes of EMCI^48–52^.

We used a relatively low number of participants (N = 110) in our study as compared to methods that require hundreds of datasets, such as those based on deep neural networks^36,38^. Requiring low number of participants to train the system is an advantage as it can be applied to smaller databases, which increases practicality of the method. For example, relying on a low number of participants enables researchers and clinicians to build their own databases or transfer results of the larger databases easier to their particular settings^56,57^. Having higher number of participants, however, brings in the advantage of generalisation that might be more difficult to achieve in smaller databases. Therefore, while the algorithm was successful with lower number of participants, it could benefit from more data and subsequently achieve a higher performance accuracy.

While publicly available large databases are becoming more common, not all such databases are suitable for all classification methods. Additionally, there is a wide variability between different databases in terms of population, and neuroimaging methods and parameters, which reduces reproducibility^53–55^. These impede translation from one database to other, or from one database to specific population in question. Therefore, it is important to test the models on more than one dataset to study sensitivity of the algorithm to different characteristics of the dataset.

We used three pipelines for segmentation of the whole brain (CAT and volBrain) and hippocampus (HIPS). All these pipelines are fully automated with minimum customisation. Therefore, it is possible to run the model without much manual handling of the data. This is an important feature as other processing methods of segmentation (e.g., FreeSurfer toolbox^58^) or more diverse methods such as connectivity analysis (e.g., as in CONN toolbox^59^) require more adjustments. Hence, there is no need for particular expertise to use these tools, which makes them more accessible and more practical in clinical settings. The validity of these methods, however, is still to be fully studied^17,26,60–62^. In particular, their level of accuracy in segmentation of brains with different disorders (such as those with atrophy) is less clear. For example, while BrainSuite toolbox^63^ has been successfully used in the past in many applications^64–66^, it is less robust against brain atrophy and major structural changes.

For feature selection, we used statistical analysis and EA methods. Statistical analysis showed very limited evidence of differences between the two groups. This indicates that the volumetric difference between EMCI and CN is quite minute and more advanced diagnosis methods are required to classify the images. The EA methods, however, showed superior performance with more than 10% improvement in the classification accuracy. NSGA-II was the strongest method. This method is one of the emerging techniques for solving multi-disciplinary stochastic optimisation problems^30,67,68^. The superior overall performance of these methods shows the potential application of these optimisation methods in clinical settings.

It must to be noted that ADNI uses behavioural measures such as MMSE and CDR scores to classify the participants into different groups of CN, MCI, and AD^81^. Such classification methods have been challenged and there has since developed a stronger emphasis on biomarkers, such as beta-amyloid deposition^82–84^. It has been shown that such measures are better indicators of AD as well as MCI^85–87^. Therefore, some of the participants in our study might have been misclassified. For example, the behavioural differences could be due to reasons other than AD. Therefore, future research should look into replicating our model using groups of participants that are classified based on latest guidelines.

Since there is no effective treatment for AD, it is extremely important to diagnose MCI as early as possible, as it might be possible to delay its progression toward AD, particularly by indirect interventions such as increased physical activity^80^. However, it is challenging to identify EMCI because there are only mild changes in the brain structures of patients compared with brain structures of CN. Our method, however, was able to classify the images into EMCI and CN based on these small differences with very high accuracy. We compared the performances of classification method based on EA and statistical method using a single modality of T1-MRI for prediction of the early MCI. Our results showed that EA can be used effectively in medical image processing and practical clinical applications. Additionally, our results showed that biomarkers based on MRI hold promise for early detection and differential diagnosis of the early stage of AD.

## Materials and methods

### Participants

Data for a total of 110 participants were extracted from a freely available public database of the Alzheimer’s disease Neuroimaging Initiative database (ADNI) (http://adni.loni.usc.edu)^69,70^. The ADNI was launched in 2003 to test whether serial MRI, fMRI, other biological markers, and clinical and neuropsychological assessments can be combined to measure the progression of MCI and early Alzheimer’s disease (AD). The Principal Investigator of ADNI is Michael W. Weiner, MD, VA Medical Center and University of California San Francisco. Enrolled participants were between 55 and 90 (inclusive) years of age, had a study partner able to provide an independent evaluation of functioning, and spoke either English or Spanish. All participants were willing and able to undergo all test procedures including neuroimaging and agreed to longitudinal follow up. All participants or their study partner gave written informed consent in line with the Declaration of Helsinki. The study protocol was approved by the local ethics committee in the VA Medical Center and University of California San Francisco and all methods were performed in accordance with relevant guidelines and regulations. See Table 3 for the details of the data. EMCI subjects had no other neurodegenerative diseases except MCI. CN subjects had no history of cognitive impairment, head injury, major psychiatric disease, or stroke. The EMCI participants were recruited with memory function approximately 1.0 SD below expected education adjusted norms^71^.

**Table 3:** Demographics of the participants.

	EMCI	CN	p
n	54	56	
Female (n [%])	26 [54.28]	27 [52.77]	
Age (mean [SD])	70.64 [6.74]	69.17 [5.82]	0.304
MMSE (mean [SD])	28.85 [1.74]	28.27 [4.65]	0.356
CDR	0.5 or 1	0	< 0.001

*CDR* clinical dementia rating, *MMSE* mini-mental state exam, *CN* cognitively normal, *EMCI* early mild cognitive impairment.

### Proposed method

**Figure 3:**
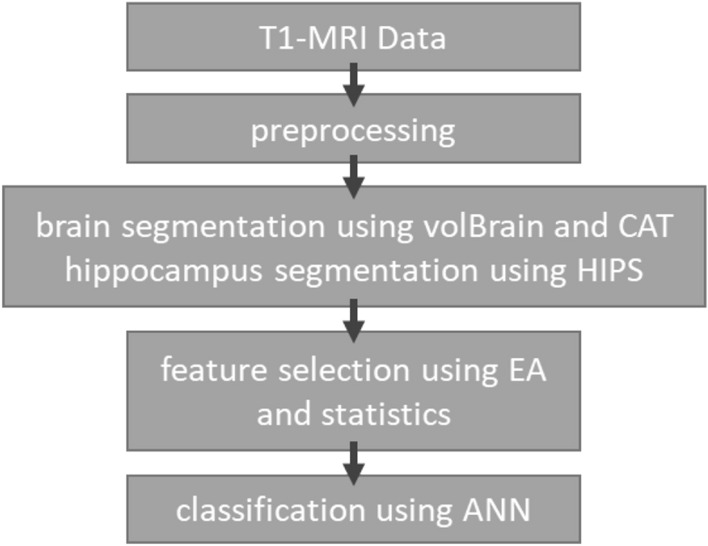
The procedure of the proposed method. Five evolutionary algorithms (EA) were used: genetic algorithm (GA), nondominated sorting genetic algorithm II (NSGA-II), ant colony optimisation (ACO), simulated annealing (SA) and particle swarm optimisation (PSO).

The procedure of our proposed method is shown in Fig. 3. In this method, T1-MRI data of healthy participants (CN; n = 56) and patients (EMCI; n = 54) are extracted from the ADNI database. A similar segmentation method was used as in an earlier report^26^, see also Supplementary Methods section. We obtained volumes of different brain areas using volBrain, CAT and HIPS. These methods are based on an advanced pipeline providing automatic segmentation of different brain structures from T1-weighted MRI. Preprocessing and segmentation of brain areas are done using volBrain and CAT for the whole brain, and HIPS for subfields of the hippocampus. Subsequently, the extracted features are given to one of the optimisation methods to select the best subset of parameters in conjunction with the classification method. Optimisation methods consisted of PSO and statistical algorithms. The outputs of these methods are given to an ANN with three hidden layers to classify the data into CN and EMCI.

### Multiobjective optimisation algorithm

A similar algorithm was used as in an early publication^72^. Nondominated sorting genetic algorithm II (NSGA-II) has recently been shown to be an effective method of optimisation. NSGA-II is a fast and superior method based on genetic algorithm to solve multiobjective optimisation problems to capture a number of solutions simultaneously^67^. In this algorithm, nondomination is used as ranking criterion of solution, and fitness sharing is used for diversification control in the search space. All the operators in genetic algorithms (i.e., selection, crossover and mutation) are also used here. NSGA-II uses binary features to fill a mating poll. Crossover and mutation operators are applied to certain portions of the mating pool members. Original, offspring, and mutant populations are merged to create a larger one. Nondomination and crowding distance are used to sort the new members. A specific number of individuals in the sorted population are transferred to the next generation. Certain number of individuals in the sorted population is selected and others are excluded. This cycle iterates until stop conditions are satisfied. The conventional NSGA has a computational complexity of $O({MN}^{3})$, where $M$ is the number of objectives and $N$ is the population size. NSGA-II, on the other hand, has an overall complexity $O\left({MN}^{2}\right)$, which is significantly lower than other GAs^67^. In dealing with multiobjective problems, designer may be interested in a set of pareto-optimal points, instead of a single point. After termination of the optimisation process, nondominated solutions form the Pareto frontier. Each of the solutions on the Pareto frontier can be considered as an optimal strategy for a specific situation^73–75^. In this study, the mutation percentage and mutation rate were set to 0.4 and 0.1, respectively; population size was 25 equal to the mating pool size, and crossover percentage was 14%.

In addition, we used four other EA methods: genetic algorithm (GA), ant colony optimisation (ACO), simulated annealing (SA) and particle swarm optimisation (PSO). For further details see Supplementary Method document.

### Classification method

For the classification of EMCI and CN, we used a multi-layer perceptron artificial neural network (ANN) with three fully-connected hidden layers with 20, 10 and 5 nodes each, respectively^72^. The classification method was performed via an 80/20 split; 80% of the data was used for the training and 20% of the data was used for validation. We used Levenberg–Marquardt Back propagation (LMBP) algorithm for training and mean square error as a measure of performance^76–78^. The LMBP has three steps (1) propagate the input forward through the network; (2) propagate the sensitivities backward through the network from the last layer to the first layer; and finally (3) update the weights and biases using Newton’s computational method^79^. In the LMBP algorithm the performance index $F\left(x\right)$ is formulated as:

$$F\left(x\right)={e}^{T}\left(x\right)e\left(x\right)$$

where $e$ is a vector of network error, and $x$ is the vector matrix of network weights and biases. The network weights are updated using the Hessian matrix and its gradient:

$${x}_{k+1}={x}_{k}-{\left({J}^{T}J+\mu I\right)}^{-1}{J}^{T}e={x}_{k}-{\left(\mathrm{H}+\mu I\right)}^{-1}\mathrm{G}$$

where $J$ represent Jacobian matrix. The Hessian matrix $H$ and its gradient $G$ are calculated using:

$$H={J}^{T}J$$

$$G={J}^{T}e$$

where the Jacobian matrix is calculated by:

$$J={S}^{m}{a}^{m-1}$$

where ${a}^{m-1}$ is the output of the $(m-1)\mathrm{th}$ layer of the network, and ${S}^{m}$ is the sensitivity of $F\left(x\right)$ to changes in the network input element in the $m{\text{th}}$ layer and is calculated by:

$${S}^{m}={F}^{m}({n}^{m})({w}^{m+1}){S}^{m+1}$$

where $${w}^{m+1}$$ represents the neuron weight at ($$m+1)$$th layer, and $$n$$ is the network input^79^.

## Supplementary Information

Supplementary Figures.

Supplementary Tables.

Supplementary Methods.

## Data Availability

No datasets were generated or analysed during the current study.
