# Supplementary material for: Diagnosis of early mild cognitive impairment using a multiobjective optimization algorithm based on T1-MRI data
Source: Sci Rep. 2022 Jan 19;12:1020. doi: 10.1038/s41598-022-04943-3 (PMC8770462; doi:10.1038/s41598-022-04943-3)
Supplement: Supplementary file 1 — Supplementary Figures. [file 41598_2022_4943_MOESM1_ESM.docx]

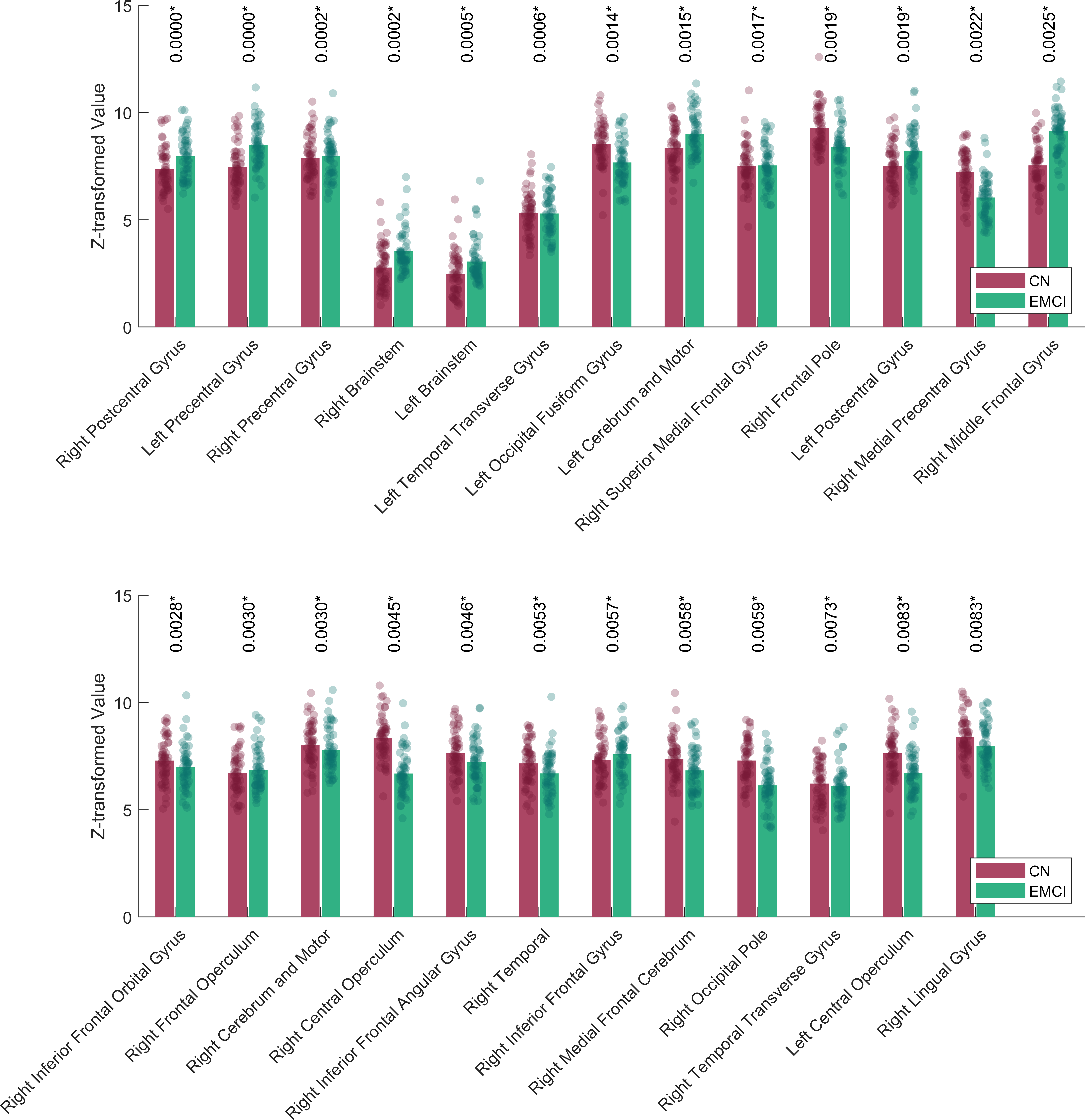


Supplementary Figure 1. Statistical comparison of brain areas segmented using CAT. *p < 0.05 FDR corrected for multiple comparison (p < 0.0094). See Supplementary Table 1 for further details.


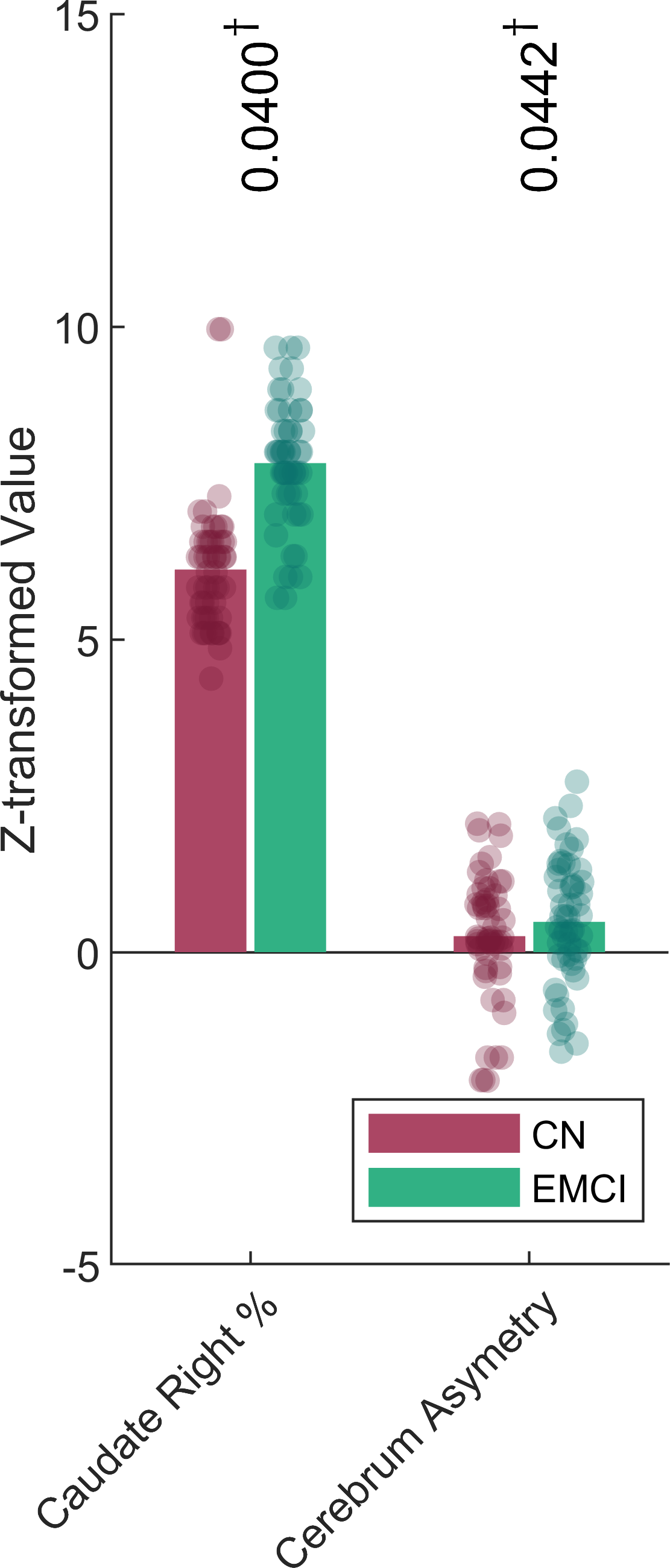


Supplementary Figure 2. Statistical comparison of brain areas segmented using volBrain. ^†^p < 0.05. No brain areas survived FDR correction for multiple comparison. See Supplementary Table 2 for further details.


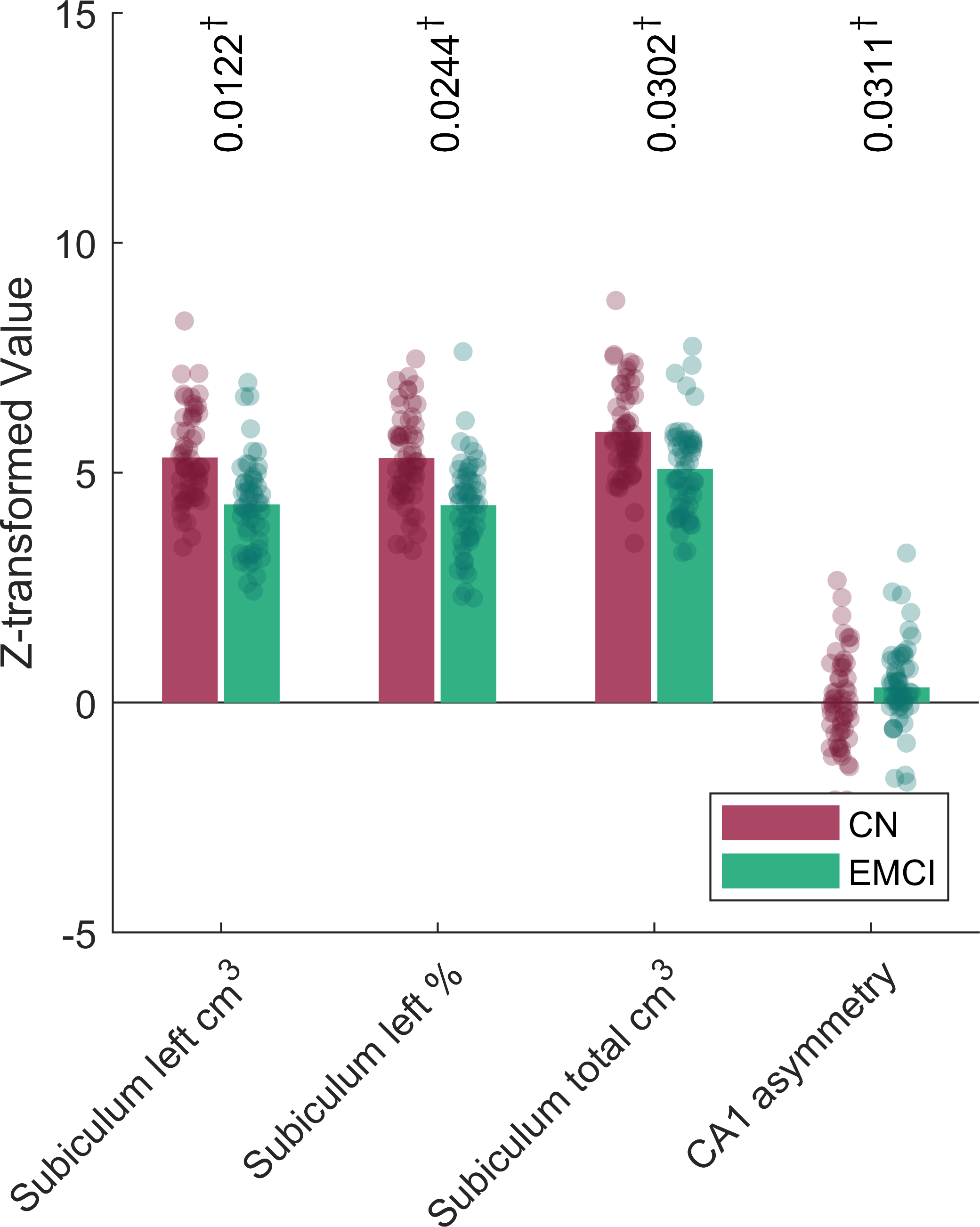


Supplementary Figure 3. Statistical comparison of brain areas segmented using HIPS. ^†^p < 0.05. No brain areas survived FDR correction for multiple comparison. See Supplementary Table 3 for further details.


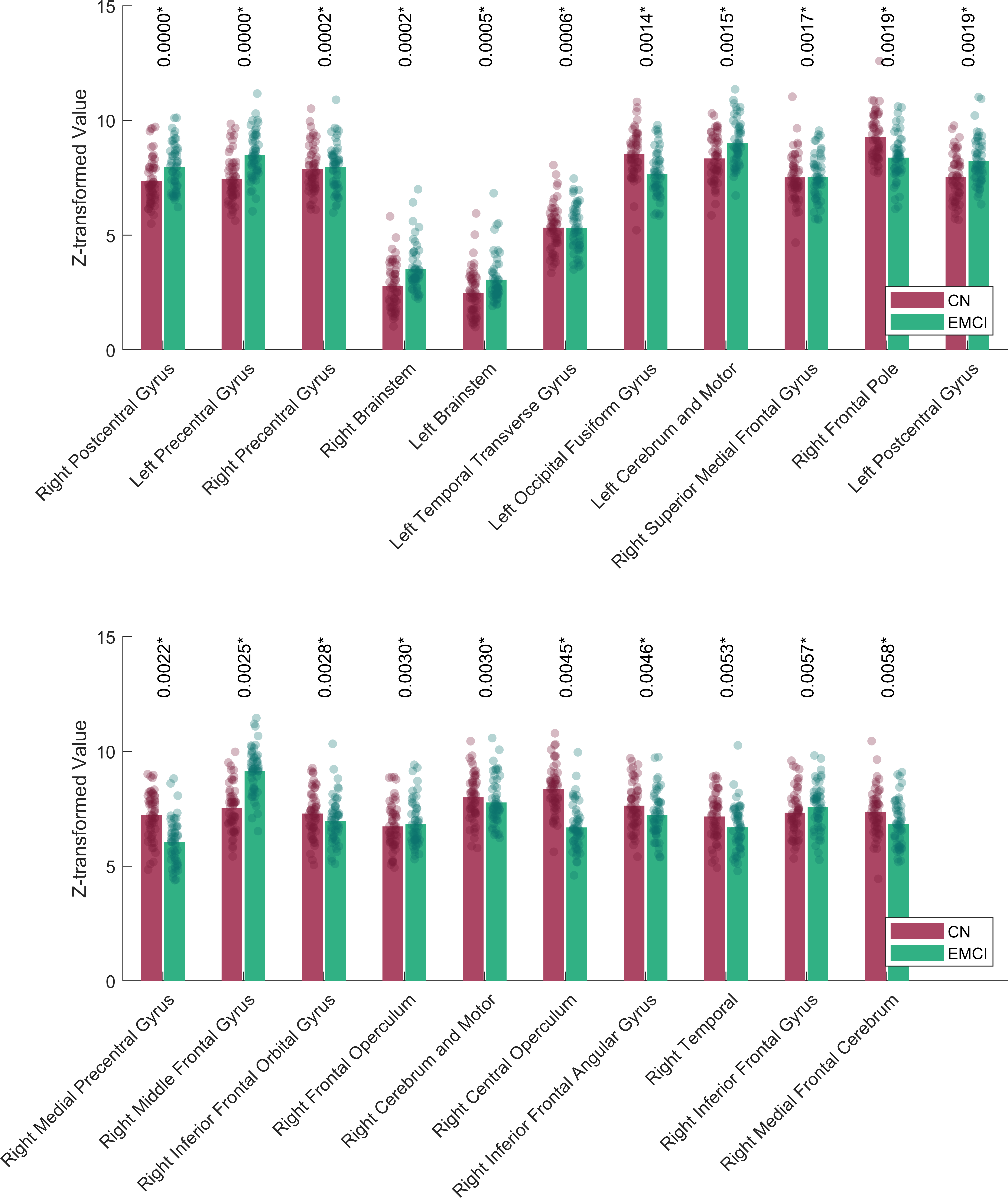


Supplementary Figure 4. Statistical comparison of brain areas segmented using CAT and HIPS. *p < 0.05 FDR corrected for multiple comparison (p < 0.0094)


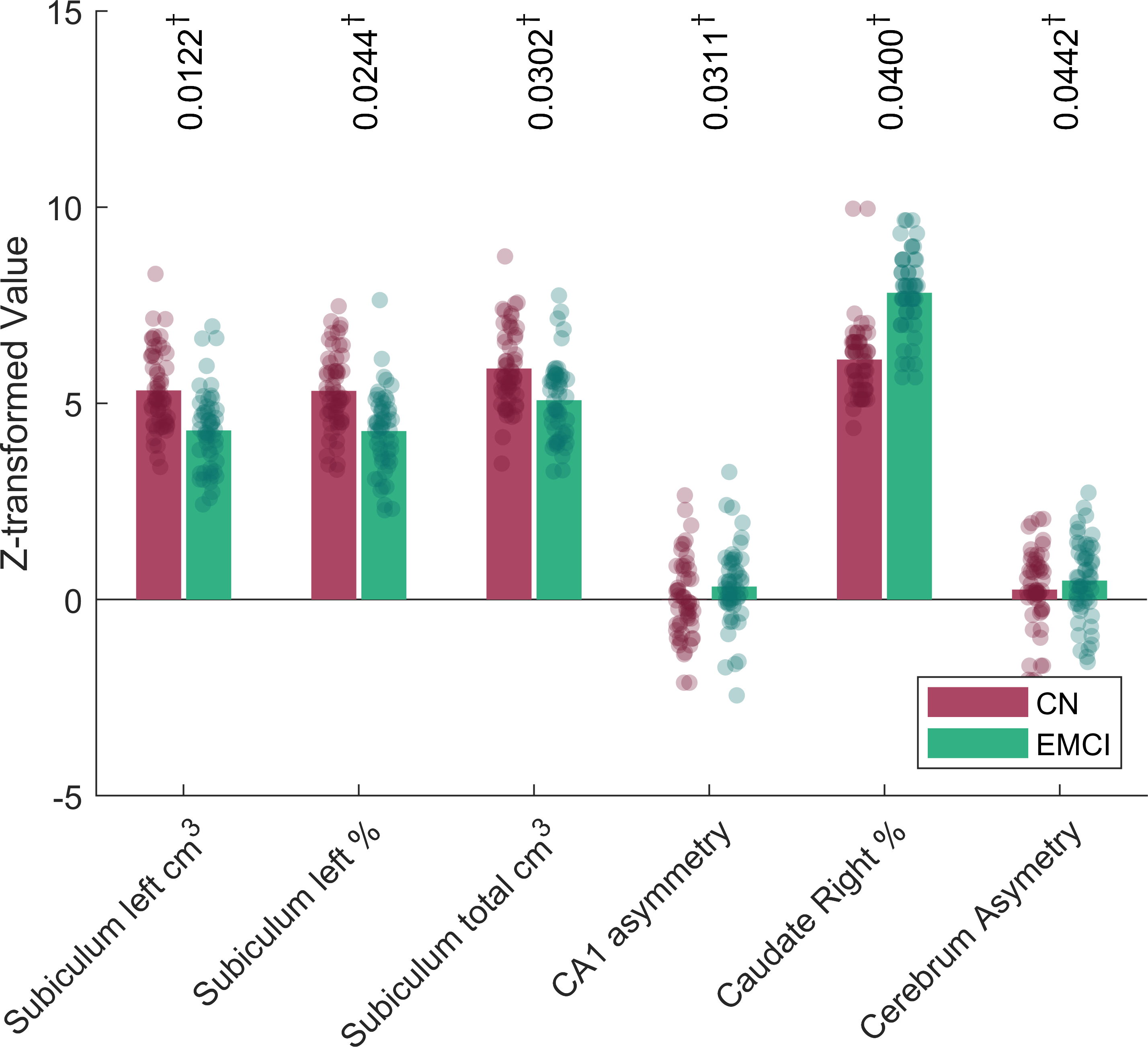


Supplementary Figure 5. Statistical comparison of brain areas segmented using volBrain and HIPS. ^†^p < 0.05. No brain areas survived FDR correction for multiple comparison.
