# Supplementary material for: Diagnosis of early mild cognitive impairment using a multiobjective optimization algorithm based on T1-MRI data
Source: Sci Rep. 2022 Jan 19;12:1020. doi: 10.1038/s41598-022-04943-3 (PMC8770462; doi:10.1038/s41598-022-04943-3)
Supplement: Supplementary file 2 — Supplementary Tables. [file 41598_2022_4943_MOESM2_ESM.docx]

**Supplementary Table 1.** Independent sample t-tests, comparing the volume of different segments between EMCI and HC using CAT. Brain areas are sorted based on the p-values of the Right hemisphere.

| Area | Right↑ | Left | Area | Right | Left |
| --- | --- | --- | --- | --- | --- |
| Postcentral Gyrus | <.0001* | 0.0019* | Posterior Cingulate Gyrus | 0.0481^†^ | 0.0516 |
| Precentral Gyrus | <.0001* | 0.0002* | Anterior Cingulate Gyrus | 0.0495^†^ | 0.8788 |
| Brainstem | 0.0002* | 0.0005* | Middle Temporal Gyrus | 0.0495^†^ | 0.1222 |
| Temporal Transverse Gyrus | 0.0006* | 0.0073* | Temporal Pole | 0.0546 | 0.3619 |
| Occipital Fusiform Gyrus | 0.0014* | 0.0106^†^ | Medial Orbital Gyrus | 0.0561 | 0.5050 |
| Cerebrum and Motor | 0.0015* | 0.0030* | Subcallosal Area | 0.0569 | 0.0947 |
| Superior Medial Frontal Gyrus | 0.0017* | 0.1854 | Superior Occipital Gyrus | 0.0642 | 0.1389 |
| Frontal Pole | 0.0019* | 0.0110^†^ | Angular Gyrus | 0.0660 | 0.1488 |
| Medial Precentral Gyrus | 0.0022* | 0.0297^†^ | Thalamus Proper | 0.0669 | 0.1854 |
| Middle Frontal Gyrus | 0.0025* | 0.0366^†^ | Middle Cingulate Gyrus | 0.0783 | 0.3052 |
| Inferior Frontal Orbital Gyrus | 0.0028* | 0.0383^†^ | Lateral Ventricle | 0.0878 | 0.9738 |
| Frontal Operculum | 0.0030* | 0.0430^†^ | Parahippocampus Gyrus | 0.0912 | 0.2039 |
| Central Operculum | 0.0045* | 0.0083* | Cuneus | 0.0983 | 0.1471 |
| Inferior Frontal Angular Gyrus | 0.0046* | 0.0642 | Inferior Temporal Gyrus | 0.1645 | 0.3682 |
| Temporal | 0.0053* | 0.0743 | CLCVL VI-VII | 0.1814 | 0.6303 |
| Inferior Frontal Gyrus | 0.0057* | 0.3253 | Pallidum | 0.1854 | 0.1955 |
| Medial Frontal Cerebrum | 0.0058* | 0.0651 | Exterior Cerebellum | 0.2752 | 0.6260 |
| Occipital Pole | 0.0059* | 0.0166^†^ | Superior Temporal Gyrus | 0.2778 | 0.3342 |
| Lingual Gyrus | 0.0083* | 0.0316^†^ | Inferior Lateral Ventricle | 0.3166 | 1.0000 |
| Inferior Occipital Gyrus | 0.0094* | 0.2306 | Third Ventricle | 0.3494 | 0.3746 |
| Planum Polare | 0.0094* | 0.0166^†^ | Anterior Orbital Gyrus | 0.4110 | 0.4898 |
| Precuneus | 0.0158^†^ | 0.0326^†^ | Entorhinal Area | 0.4282 | 0.5088 |
| Posterior Insula | 0.0164^†^ | 0.0259^†^ | Putamen | 0.4823 | 0.8319 |
| Parietal Operculum | 0.0166^†^ | 0.0276^†^ | Optic Chiasm | 0.5088 | 1.0000 |
| Superior Frontal Gyrus | 0.0175^†^ | 0.0753 | Fourth Ventricle | 0.5360 | 0.6345 |
| Fusiform Gyrus | 0.0183^†^ | 0.0634 | Basal Cerebrum & Forebrain Brain | 0.5843 | 0.9833 |
| Calcarine and Cerebrum | 0.0202^†^ | 0.1471 | Cerebral White Matter | 0.6009 | 0.6303 |
| Gyrus Rectus | 0.0218^†^ | 0.2522 | Accumbens | 0.6134 | 0.6690 |
| Anterior Insula | 0.0240^†^ | 0.1326 | Hippocampus | 0.6218 | 0.6909 |
| Medial Postcentral Gyrus | 0.0272^†^ | 0.0280^†^ | CLCVL VIII-X | 0.6517 | 0.9072 |
| Middle Occipital Gyrus | 0.0289^†^ | 0.2306 | CLCVL I-V | 0.6953 | 0.8977 |
| Superior Parietal Lobule | 0.0297^†^ | 0.0609 | Caudate | 0.8226 | 0.9642 |
| Supramarginal Gyrus | 0.0302^†^ | 0.0867 | Cerebellum White Matter | 0.9167 | 0.9214 |
| Posterior Orbital Gyrus | 0.0377^†^ | 0.1266 | Amygdala | 0.9690 | 0.9738 |
| Ventral Ventricle | 0.0418^†^ | 0.1488 | CSF | 0.4247 | 0.7994 |
| Lateral Orbital Gyrus | 0.0436^†^ | 0.1358 |  |  |  |

Notes: WM = white matter, CSF = cerebrospinal fluid, CLCVL: Cerebellar Lobule Cerebellar Vermal Lobules; **p* < 0.05 FDR correction for multiple comparisons (*p* ≤ 0.0094); ^†^ *p* < 0.05 but not surviving FDR correction for multiple comparisons.

**Supplementary Table 2.** Independent sample t-tests, comparing the volume of different segments between EMCI and HC using volBrain. Brain areas are sorted based on the p-values of the total area.

|  | **Total↑** |  | **Left** |  | **Right** |  |  |
| --- | --- | --- | --- | --- | --- | --- | --- |
|  | **%** | **cm^3^** | **%** | **cm^3^** | **%** | **cm^3^** | **Asym.** |
| Cerebellum | 0.0753 | 0.8694 | 0.0773 | 0.8366 | 0.0753 | 0.8694 | 0.3975 |
| Caudate | 0.0971 | 0.4639 | 0.2153 | 0.5539 | 0.0400^†^ | 0.3108 | 0.4898 |
| Hippocampus | 0.1165 | 0.4712 | 0.1540 | 0.2049 | 0.0715 | 0.0773 | 0.2169 |
| Cerebellum GM | 0.2996 | 0.5088 | 0.2622 | 0.5360 | 0.2648 | 0.6690 |  |
| Putamen | 0.3549 | 0.5925 | 0.2659 | 0.4423 | 0.6074 | 0.8087 | 0.1208 |
| Brainstem | 0.3843 | 0.6092 |  |  |  |  |  |
| Amygdala | 0.4600 | 0.2610 | 0.2770 | 0.1110 | 0.9416 | 0.5321 | 0.1591 |
| Lateral ventricles | 0.4749 | 0.5802 | 0.8670 | 0.9976 | 0.2472 | 0.3108 | 0.0901 |
| Thalamus | 0.5157 | 0.3778 | 0.6714 | 0.3556 | 0.5616 | 0.3650 | 0.1389 |
| Cerebrum GM | 0.5243 | 0.1266 | 0.5599 | 0.1357 | 0.4247 | 0.0971 |  |
| Cerebellum WM | 0.5802 | 0.4458 | 0.3682 | 0.2995 | 0.8883 | 0.6431 |  |
| Accumbens | 0.6138 | 0.9928 | 0.4715 | 0.7513 | 0.2211 | 0.8883 | 0.4458 |
| Tissue WM | 0.7175 | 0.5761 |  |  |  |  |  |
| Cerebrum WM | 0.7994 | 0.5967 | 0.9119 | 0.6560 | 0.7131 | 0.5165 |  |
| Tissue GM | 0.8272 | 0.1296 |  |  |  |  |  |
| Tissue Brain | 0.8459 | 0.3494 |  |  |  |  |  |
| Tissue CSF | 0.8459 | 0.5165 |  |  |  |  |  |
| Globus Pallidus | 0.8499 | 0.4712 | 0.9034 | 0.2169 | 0.8506 | 0.6560 | 0.1591 |
| Cerebrum | 0.9881 | 0.3080 | 0.9738 | 0.3587 | 0.8647 | 0.2674 | 0.0442^†^ |
| Tissue IC | 1.0000 | 0.1776 |  |  |  |  |  |

Notes: GM = gray matter, WM = white matter, CSF = cerebrospinal fluid, IC = internal capsule, ^†^ *p* < 0.05 but not surviving FDR correction for multiple comparisons.

**Supplementary Table 3.** Independent sample t-tests, comparing the volume of different segments between EMCI and HC using HIPS. Brain areas are sorted based on the p-values of the total area.

|  | **Total** |  | **Left** |  | **Right** |  |  |
| --- | --- | --- | --- | --- | --- | --- | --- |
|  | **%** | **cm^3^** | **%** | **cm^3^** | **%** | **cm^3^** | **Asym.** |
| Subiculum | 0.0600 | 0.0302^†^ | 0.0244^†^ | 0.0122^†^ | 0.3572 | 0.2237 | 0.1179 |
| CA4-DG | 0.3525 | 0.1097 | 0.5599 | 0.3572 | 0.2522 | 0.0705 | 0.4936 |
| Hippocampus | 0.3746 | 0.4712 | 0.4178 | 0.5761 | 0.3195 | 0.4178 | 0.6997 |
| CA2-CA3 | 0.3876 | 0.6431 | 0.2522 | 0.3052 | 0.7423 | 0.8226 | 0.1311 |
| SR-SL-SM | 0.4352 | 0.2996 | 0.4042 | 0.3556 | 0.4178 | 0.2147 | 0.0878 |
| CA1 | 0.5419 | 0.2497 | 0.8835 | 0.4823 | 0.3372 | 0.1405 | 0.0311^†^ |

Notes: SR-SL-SM = strata radiatum/lacunosum/moleculare. ^†^ *p* < 0.05 but not surviving FDR correction for multiple comparisons.

**Supplementary Table 4.** Independent sample t-tests, comparing the volume of different segments between EMCI and HC using HIPS. Brain areas are sorted based on the p-values of the total area.

| Methods | Pipelines | Accuracy | Methods | Pipelines | Accuracy |
| --- | --- | --- | --- | --- | --- |
| NSGA2 | CAT | 95% | ACO | CAT | 94% |
|  | VolBrain | 96% |  | VolBrain | 93% |
|  | HIPS | 91% |  | HIPS | 89% |
|  | HIPS + CAT | 97% |  | HIPS + CAT | 96% |
|  | HIPS + VolBrain | 98% |  | HIPS + VolBrain | 95% |
| GA | CAT | 94% | SA | CAT | 94% |
|  | VolBrain | 95% |  | VolBrain | 95% |
|  | HIPS | 92% |  | HIPS | 93% |
|  | HIPS + CAT | 96% |  | HIPS + CAT | 96% |
|  | HIPS + VolBrain | 96% |  | HIPS + VolBrain | 97% |
| PSO | CAT | 95% | Statistical | CAT | 91% |
|  | VolBrain | 96% |  | VolBrain | 90% |
|  | HIPS | 90% |  | HIPS | 88% |
|  | HIPS + CAT | 97% |  | HIPS + CAT | 93% |
|  | HIPS + VolBrain | 97% |  | HIPS + VolBrain | 92% |

**Supplementary Table 5.** Demographics of the participants in the AD condition.

| n | 54 |
| --- | --- |
| Female (n [%]) | 26 [48] |
| Age (mean[SD]) | 72.74 [6.58] |
| MMSE | 22.5 [1.85] |
| CDR | 0.5 or 1 |

**Supplementary Table 6.** Accuracy of different optimisation and the segmentation methods classifying the two groups of cognitively normal individuals and those with Alzheimer’s disease.

| Methods | Pipelines | Accuracy | Methods | Pipelines | Accuracy |
| --- | --- | --- | --- | --- | --- |
| NSGA2 | CAT | 95% | ACO | CAT | 94% |
|  | VolBrain | 96% |  | VolBrain | 93% |
|  | HIPS | 91% |  | HIPS | 89% |
|  | HIPS + CAT | 97% |  | HIPS + CAT | 96% |
|  | HIPS + VolBrain | 98% |  | HIPS + VolBrain | 95% |
| GA | CAT | 94% | SA | CAT | 94% |
|  | VolBrain | 95% |  | VolBrain | 95% |
|  | HIPS | 92% |  | HIPS | 93% |
|  | HIPS + CAT | 96% |  | HIPS + CAT | 96% |
|  | HIPS + VolBrain | 96% |  | HIPS + VolBrain | 97% |
| PSO | CAT | 95% | Statistical | CAT | 91% |
|  | VolBrain | 96% |  | VolBrain | 90% |
|  | HIPS | 90% |  | HIPS | 88% |
|  | HIPS + CAT | 97% |  | HIPS + CAT | 93% |
|  | HIPS + VolBrain | 97% |  | HIPS + VolBrain | 92% |
