# Supplementary material for: Diagnosis of early mild cognitive impairment using a multiobjective optimization algorithm based on T1-MRI data
Source: Sci Rep. 2022 Jan 19;12:1020. doi: 10.1038/s41598-022-04943-3 (PMC8770462; doi:10.1038/s41598-022-04943-3)
Supplement: Supplementary file 3 — Supplementary Methods. [file 41598_2022_4943_MOESM3_ESM.docx]

Supplementary Methods

# Segmentation Methods

Similar segmentation methods were used as a previous publication ^1^.

## HIPS and volBrain

The volumes of Cerebrospinal fluid (CSF), white matter (WM), grey matter (GM), brain hemispheres, cerebellum and brainstem were obtained using volBrain pipeline ^2^. This method is based on an advanced pipeline providing automatic segmentation of diﬀerent brain structures from T1 weighted MRI, Figure 1. The preprocessing is based on the following procedure: (1) a denoising step with an adaptive non-local mean ﬁlter, (2) an aﬃne registration in the Montreal Neurological Institute (MNI) space, (3) a correction of the image inhomogeneities, and (4) an intensity normalisation. (5) Afterwards, MRI images are segmented in the MNI space using non-local patch-based multi-atlas method. Images were corrected for intensity inhomogeneity using the N4 algorithm ^3^, and the images were segmented into brain/non-brain using a semi-automated technique (MIDAS). The Non-Local Means ﬁlter ^4^ was applied to each pixel of the image by computing a weighted average of surrounding pixels using a robust similarity measure that takes into account the neighbouring pixels surrounding the pixel being compared. This segmentation method is based on the idea of the non-local patch-based label fusion technique, where patches of the brain image to be segmented are compared with those of the training library, looking for similar patterns within a defined search volume to assign the proper label ^5,6^. HIPS and volBrain are used for segmentation of the hippocampus subfields and the rest of the brain, respectively ^7^.


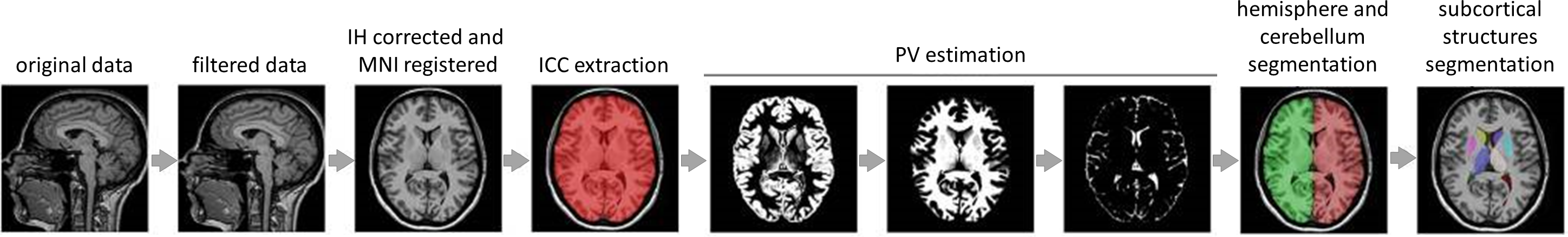


Figure 1. Processing pipeline for volBrain and HIPS adapted from Manjón and Coupé (2016) ^2^ under the terms of the Creative Commons Attribution License (CC BY).

## CAT

Computational Anatomy Toolbox (CAT) is a powerful package for brain T1-MRI data segmentation, Figure 2. It is a voxel base estimation method ^8^. The CAT preprocessing steps are as follows: (1) spatial registration to a template, (2) tissue segmentation into grey, white matter and CSF, and (3) bias correction of intensity non-uniformities. (4) Finally, segments are extracted by scaling the amount of volume changes based on spatial registration, so that the total volume of grey matter in the modulated image remains the same as the original image. For correction of the orientation and size of the brain, non-linear registration methods are applied to the image ^9^. The projection-based thickness (PBT) method was used for calculation of the cortical thickness and central surface ^9,10^. Spatial-adaptive Non-Local Means (SANLM) and classical Markov Random Field (MRF) were used for image Denoising ^11^. Adaptive Maximum a Posterior (AMAP) method was used for segmentation ^8^.


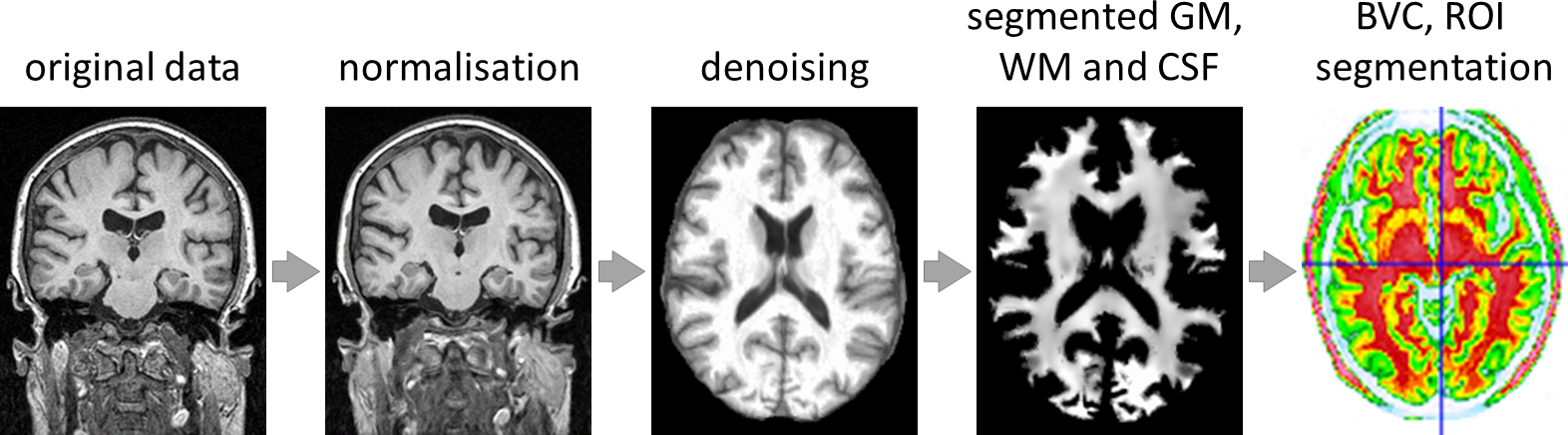


Figure 2. Processing pipeline for CAT.

## BrainSuite

BrainSuite is an open-source software tool that enables largely automated cortical surface extraction from MRI of the brain, Figure 3. BrainSuite includes automatic cortical surface extraction, bias field correlation, cerebrum labelling, and surface generation features. In addition, this toolbox is used in tractography and connectivity matrix calculation in diffusion imaging data ^12^.


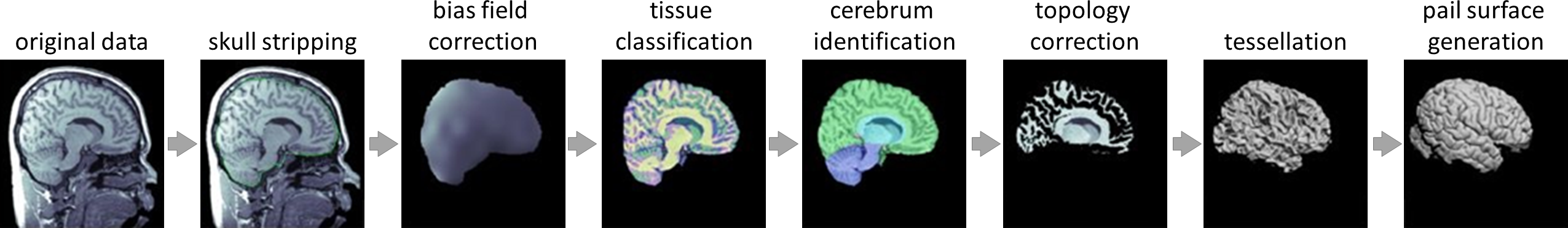


Figure 3. Processing pipeline for BrainSuite.

# Optimisation Methods

Similar algorithms were used as in an early publication ^13^.

## Genetic Algorithms (GA)

GA is one of the most advanced algorithms for feature selection ^14^. This algorithm is based on the mechanics of natural genetics and biological evolution for finding the optimum solution. It consists of five steps: selection of initial population, evaluation of fitness function, pseudo-random selection, crossover, and mutation ^15^. The procedure of GA consists of the following four steps ^16^:

1. *Individual encoding*: Each individual is encoded as a binary vector of size $P$, where the entry $b_{i}=1$ states for the predictor $p_{i}$ that is defined for that individual, $b_{i}=0$ if the predictor $p_{i}$ is not included in that particular individual ($i=1,\ldots,P$).
2. *Initial population*: Given the binary representation of the individuals, the population is a binary matrix where its rows are the randomly selected individuals, and the columns are the available predictors. An initial population with a predefined number of individuals is generated with a random selection of 0 and 1 for each entry.
3. *Fitness function*: the fitness value of the individual in the population is calculated using predefined fitness function. Individuals with the lowest prediction error and fewer predictors have been selected for next generation.
4. *Genetic operators*: applying genetic operators to create the next generation.

The genetic operators are, *Selection* (random selection of members based on their fitness value; fitter members are more likely to be chosen), *Crossover* (the new generation is created by exchanging elements between two selected parents from the previous step), *Mutation* (elements in a selected member is changed), and *Stop Criteria* (the criteria to indicate the end of the search) ^16^. In our study, we used roulette wheel selection for selection of the possible valuable solutions to producing offsprings for the next generation. Single point, double point, and uniform crossover methods are used to generate new members. In this study we used 0.3 and 0.1 as mutation percentage and mutation rate, respectively; 20 members per population, crossover percentage was 14 with 8 as selection pressure ^17,18^.

## Ant colony optimisation algorithm (ACO)

ACO is a metaheuristic optimisation method based on the behaviour of ants ^19^. This algorithm consists of four steps: initialisation, creation of ant solutions (a set of ants build a solution to the problem being solved using pheromones values and other information), local search (improvement of the created solution by ants), and global pheromone update (update in pheromone variables based on search action followed by ants) ^20^. ACO requires a problem to be described as a graph: nodes represent features and edges indicate which features should be selected for the next generation. In features selection, the ACO tries to find the best solutions using prior information from previous iterations. The search for the optimal feature subset consists of an ant traveling through the graph with a minimum number of nodes required for satisfaction of stopping criterion ^21^. See Figure 4 for the procedure of the traverse of an ant placed at node $a$. This ant has a choice of which feature to add next to its path (dotted lines). It traverses through the graph to find a path that satisfies the stopping criterion (e.g., a suitably high classification accuracy has been achieved with this subset). In this example, the ant chooses next feature $b$ based on a set of transition rules, then $c$ and then $d$. Upon arrival at$d$, the current subset $\{a;b;c;d\}$ is determined to satisfy the traversal stopping criterion. At termination of search, the algorithm outputs this feature subset as a candidate for data reduction ^22^.


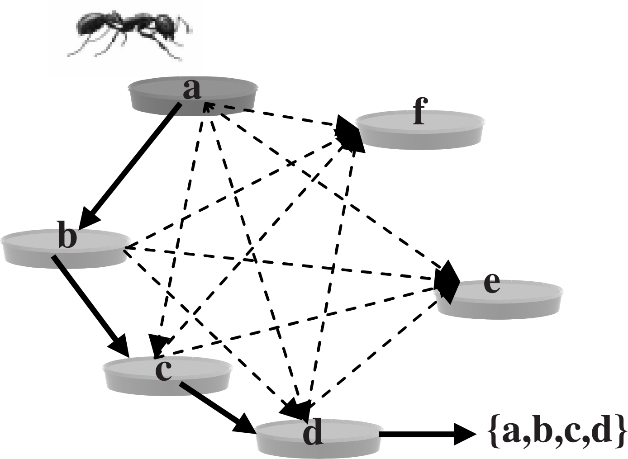


Figure 4. A sample example of ant traveling through multiple features in ant colony optimisation algorithm (ACO). Here feature subset of $\{a;b;c;d\}$ is selected as a possible solution ^22^.

The probability of an ant at feature $i$ choosing to travel to feature $j$ at time $t$:

$$p_{ij}^{n}\left( t \right)=\left\{ \begin{matrix} \frac{\left[ \vartheta_{ij}\left( t \right) \right]^{\alpha}.\left[ \varphi_{ij} \right]^{\beta}}{\sum_{l\in J_{i}^{n}} \left[ \vartheta_{ij}\left( t \right) \right]^{\alpha}.\left[ \varphi_{ij} \right]^{\beta}} & \text{if} j\in J_{i}^{n} \\ 0 & \text{otherwise} \end{matrix} \right.$$

where $n$ is the number of ants, $\varphi_{ij}$ is the heuristic desirability of choosing feature $j$ when at feature $i$, $J_{i}^{n}$ is the set of nodes next to node $i$, which have not yet been visited by the ant $n$. The $\alpha>0$ and $\beta>0$ are two parameters that determine the relative importance of the pheromone value and heuristic information, respectively, and $\vartheta_{ij}$is the amount of virtual pheromone on edge $(i,j)$. The pheromone on each edge is updated according to the following formula ^22^:

$$\vartheta_{ij}\left( t+1 \right)=\left( 1-\rho\right)\vartheta_{ij}\left( t \right)+\rho\Delta\vartheta_{ij}\left( t \right)$$

$$\Delta\vartheta_{ij}\left( t \right)=\sum_{n=1}^{N} (\gamma(F^{n})/\left| F^{n} \right|)$$

This is the case if the edge $(i,j)$ has been traversed; $\Delta\vartheta_{ij}\left( t \right)$ is 0 otherwise. The value $0\leq\rho\leq1$ is decay constant used to simulate the evaporation of the pheromone. The pheromone is updated according to both the measure of the "goodness" of the ant's feature subset $\gamma$ and the size of the subset itself. By this definition, all ants update the pheromone ^22^. $F^{n}$ is the feature subset found by ant $n$. We used 10, 0.05, 1, 1 and 1 for the number of ants, evaporation rate, initial weight, exponential weight, and heuristic weight, respectively.

## Simulated annealing (SA)

SA is a stochastic search algorithm, which is particularly useful in large-scale linear regression models ^23^. In this algorithm, the new feature subset is selected entirely at random based on the current state. After an adequate number of iterations, a dataset can be created to quantify the difference in performance with and without each predictor ^24,25^. SA utilises a certain probability to accept a worse solution. The algorithm starts with a randomly generated solution; in each iteration, a neighbour solution to the best solution so far is generated according to a predefined neighbourhood structure and evaluated using a fitness function. The improving move is accepted, whilst worse neighbours are accepted with a certain probability determined by the Boltzmann probability, $P=e-\theta/ T$ where $\theta$ is the difference between the fitness of the best solution and the generated neighbour. Moreover, $T$ is a temperature, which periodically decreases during the search process according to a certain cooling schedule. First, the current temperature $T$ is set to be a very large number ^26,27^. We set initial temperature and temperature reduction rate with 10 and 0.99, respectively.

## Particle swarm optimisation (PSO)

PSO is a stochastic optimisation method based on the behaviour of swarming animals such as birds and fish. Each member finds optimal regions of the search space by coordinating with other members of the population. In this method, each possible solution is represented as a particle with a certain position and velocity moving through the search space ^28–30^. In a PSO with an N-dimensional search space, the particle position and velocity are formulated by:

$$V_{ij}=\left| w\times x_{ij}+c_{p}\times r_{p}\times\left( p_{ij}-x_{ij} \right)+c_{g}\times r_{g}\times\left( p_{gj}-x_{ij} \right) \right|$$

$x_{ij}=x_{ij}+v_{ij}$,

where $V_{i}$ and $x_{i}$ refer to the velocity and position of the particle $i$, respectively, and $j$, ranging from 1 to N (total number of features). $c_{p}$ is the so-called cognitive parameter, defining the degree of acceleration towards the particles’ individual local best position $p_{ij}$. $c_{g}$ is a social parameter, defining the acceleration towards the global best position $p_{gj}$. $w$ is an inertia parameter, regulating the overall rate of change. The stochastic nature of the velocity equation is represented by $r_{p}$ and$r_{g}$, which are in the range [0, 1]. To maintain coherence in the swarm, the maximum velocity is regulated by the parameter $v_{max}$. In standard PSO implementations, typically $v_{max}=\left| x_{max}-x_{min} \right|$. We set swarm size = 20,$c_{g}=c_{p}=1.5$ and $w=0.72$.

*Statistical Approach;* to create a baseline to compare dimension reduction methods based on PSO, we also used a statistical approach to select the features based on the statistical difference between the two groups. We compared the T1-MRI data extracted features using two independent-sample t-test analyses. Subsequently, we selected the parameters based on their sorted *p* values.

# References

1.         Zamani, J., Sadr, A. & Javadi, A. A Large-scale Comparison of Cortical and Subcortical Structural Segmentation Methods in Alzheimer’ s Disease: a Statistical Approach. *bioRxiv* (2020) doi:10.1101/2020.08.18.256321.

2.         Manjón, J. v. & Coupé, P. volBrain: An Online MRI Brain Volumetry System. *Frontiers in Neuroinformatics* **10**, 1–14 (2016).

3.         Tustison, N. J. *et al.* N4ITK: Improved N3 bias correction. *IEEE Transactions on Medical Imaging* **29**, 1310–1320 (2010).

4.         Manjón, J. v., Coupé, P., Martí-Bonmatí, L., Collins, D. L. & Robles, M. Adaptive non-local means denoising of MR images with spatially varying noise levels. *Journal of Magnetic Resonance Imaging* **31**, 192–203 (2010).

5.         Coupé, P. *et al.* Patch-based segmentation using expert priors: Application to hippocampus and ventricle segmentation. *NeuroImage* **54**, 940–954 (2011).

6.         Romero, J. E., Manjón, J. v., Tohka, J., Coupé, P. & Robles, M. NABS: Non-local automatic brain hemisphere segmentation. *Magnetic Resonance Imaging* **33**, 474–484 (2015).

7.         Romero, J. E., Coupé, P. & Manjón, J. v. HIPS: A new hippocampus subfield segmentation method. *NeuroImage* **163**, 286–295 (2017).

8.         Ashburner, J. & Friston, K. J. Unified segmentation. *NeuroImage* **26**, 839–851 (2005).

9.         Gaser, C., & Dahnke, R. *CAT-A Computational Anatomy Toolbox for the Analysis of Structural MRI Data*. *Human Brain Mapping* (2016).

10.        Dahnke, R. & Gaser, C. *Brain structural trajectories over the adult lifespan*. *Human Brain Mapping* (2017).

11.        Manjón, J. v., Coupé, P., Martí-Bonmatí, L., Collins, D. L. & Robles, M. Adaptive non-local means denoising of MR images with spatially varying noise levels. *Journal of Magnetic Resonance Imaging* **31**, 192–203 (2010).

12.        Shattuck, D. W. & Leahy, R. M. BrainSuite: An automated cortical surface identification tool. *Medical Image Analysis* **6**, 129–142 (2002).

13.        Zamani, J., Sadr, A. & Javadi, A. Evolutionary optimization in classification of early-MCI patients from healthy controls using graph measures of resting-state fMRI. *bioRxiv* (2021) doi:10.1101/2021.03.04.433989.

14.        Tsai, C. F., Eberle, W. & Chu, C. Y. Genetic algorithms in feature and instance selection. *Knowledge-Based Systems* **39**, 240–247 (2013).

15.        Goldenberg, D. E. *Genetic algorithms in search, optimization and machine learning*. (Addison Wesley, 1989).

16.        Amini, F. & Hu, G. A two-layer feature selection method using Genetic Algorithm and Elastic Net. *Expert Systems with Applications* **166**, 114072 (2021).

17.        Vandewater, L., Brusic, V., Wilson, W., Macaulay, L. & Zhang, P. An adaptive genetic algorithm for selection of blood-based biomarkers for prediction of Alzheimer’s disease progression. *BMC Bioinformatics* **16**, 1–10 (2015).

18.        Johnson, P. *et al.* Genetic algorithm with logistic regression for prediction of progression to Alzheimer’s disease. *BMC Bioinformatics* **15**, 1–14 (2014).

19.        Dorigo, M., Caro, G. di & Gambardella, L. M. Ant Algorithms for Discrete Optimization. *Artificial Life* **5**, 137–172 (1999).

20.        Akhtar, A. Evolution of Ant Colony Optimization Algorithm — A Brief Literature Review. *arXiv* (2019).

21.        Kalami Heris, S. M. & Khaloozadeh, H. Ant Colony Estimator: An intelligent particle filter based on ACO ℝ. *Engineering Applications of Artificial Intelligence* **28**, 78–85 (2014).

22.        Kanan, H. R., Faez, K. & Taheri, S. M. Feature Selection Using Ant Colony Optimization (ACO): A New Method and Comparative Study in the Application of Face Recognition System. in *Advances in Data Mining. Theoretical Aspects and Applications* vol. 4597 LNCS 63–76 (Springer Berlin Heidelberg, 2007).

23.        Kirkpatrick, S., Gelatt, C. D. & Vecchi, M. P. Optimization by Simulated Annealing. *Science* **220**, 671–680 (1983).

24.        Anily, S. & Federgruen, A. Simulated Annealing Methods With General Acceptance Probabilities. *Journal of Applied Probability* **24**, 657–667 (1987).

25.        Bertsimas, D. & Tsitsiklis, J. Simulated annealing. *Statistical Science* **8**, 10–15 (1993).

26.        Lin, S. W., Lee, Z. J., Chen, S. C. & Tseng, T. Y. Parameter determination of support vector machine and feature selection using simulated annealing approach. *Applied Soft Computing Journal* **8**, 1505–1512 (2008).

27.        Mafarja, M. M. & Mirjalili, S. Hybrid Whale Optimization Algorithm with simulated annealing for feature selection. *Neurocomputing* **260**, 302–312 (2017).

28.        Kennedy, J. & Eberhart, R. Particle swarm optimization. in *Proceedings of ICNN’95 - International Conference on Neural Networks* vol. 4 1942–1948 (IEEE, 1995).

29.        Wang, X., Yang, J., Teng, X., Xia, W. & Jensen, R. Feature selection based on rough sets and particle swarm optimization. *Pattern Recognition Letters* **28**, 459–471 (2007).

30.        Team, Y. Particle swarm optimization in MATLAB. (2015).
